# Heart rate variability and burnout risk among healthy employees in jobs involving interaction with others

**DOI:** 10.3389/fphys.2026.1908141

**Published:** 2026-08-04

**Authors:** Irina Böckelmann, Jonas Hartung, Sabine Darius, Stefan Sammito, Beatrice Thielmann

**Affiliations:** 1 Department of Occupational Medicine, Faculty of Medicine, Otto von Guericke University, Magdeburg, Germany; 2 German Air Force Centre of Aerospace Medicine, Research & Development, Cologne, Germany

**Keywords:** autonomic regulation, burnout risk, heart rate variability (HRV), high-interaction occupations, mental health, occupational health prevention

## Abstract

Interaction-intensive work is characterised by high social demands, stressful situations, and emotional regulation. It can therefore be a risk factor for high level of work-related stress and burnout. Chronic stress has been linked to changes in autonomic regulation, as reflected by heart rate variability (HRV), an objective indicator of stress. The aim of the study was to investigate the association between burnout risk and HRV in healthy employees engaged in high-interaction work. A cross-sectional analysis was conducted on 202 working adults (including nursery teachers, bank clerks and medical assistants) with an average age of 41.1 ± 11.4 years (median 40.4 years, range 22–63 years). Burnout risk was assessed using the Maslach Burnout Inventory (MBI-GS). Participants were divided into three groups according to their burnout risk level. HRV was analysed comparatively across these three groups using 24-hour recordings as well as a 6-hour analysis during the day and at night. An increased risk of burnout was observed in 5.4% of the participants in the total sample. Significant differences in HRV between the three groups with and without varying levels of burnout risk were primarily observed in the 24-hour interval (including heart rate: p = 0.039; LF n.s., HF n.s. and LF/HF: p = 0.009 in each case) as well as in the 6-hour daytime interval for the MeanRR (p = 0.002), but not at night. Correlation analyses revealed predominantly weak associations between HRV and burnout dimensions. The strongest associations were observed in the daytime interval (e.g. MBI total score with LF ρ = −0.271; p < 0.01 and with pNN50 ρ = 0.271; p < 0.01). Age in particular proved was found to be a significant influencing factor in the analysis of variance, with large effect sizes on several HRV parameters (e.g. pNN50 η² = 0.178; RMSSD η² = 0.173). Even the first results suggest that work-related stress and burnout factors in healthy employees engaged in interactive work may be reflected in subtle changes in autonomic regulation, further analysis have shown that the effects are mainly driven by age and gender, two factors that have an impact on HRV. This shows that it is important to analysis not only autonomic regulation along e.g. by measuring HRV, but also to consider influencing co-factors that have an impact on them. On total, in the examined group it was not able to show a different between persons with high and low interactive work in subtle changes in autonomic regulation.

## 1 Introduction

The mental health of employees remains a central focus of preventive occupational medicine. Burnout syndrome is one possible long-term consequence of coping with work demands and high psychological stress, as well as of persistent short-term stress effects (Heinke et al., 2011; Oliveira et al., 2011; Hyman et al., 2011; Kinzl et al., 2006; Chuang et al., 2016; Richter and Hacker, 2017). In the new edition of the International Classification of Diseases (ICD-11), burnout is described as an occupational phenomenon, rather than a mental disorder. It is conceptualised there as a work-related syndrome resulting from chronic unmanaged workplace-related stress. Burnout is characterised by emotional exhaustion, cynicism/depersonalisation, as well as a reduced sense of personal accomplishment (Maslach and Jackson, 1981). Research indicates that work-related burnout can have serious personal and professional consequences for individuals and also represents a significant economic health factor for society (Kaschka et al., 2011; Murali and Banerjee, 2018; Copur, 2019). Key risk factors for developing burnout syndrome can be found in the personal, social and work-related spheres. Personality-related individual risk factors include predisposing traits, excessive perfectionism, low self-efficacy, a strong need for harmony, belief in external control, overestimation of one’s resilience, avoidance or a lack of recovery strategies, and defining one’s personality and self-worth primarily through (work) performance (Korczak et al., 2010; Häusser et al., 2010; Ganster and Rosen, 2013; Reichhart and Pusch, 2023). Social risk factors include, for example, a lack of or insufficient social support. The identified external, work-related risk factors for burnout include excessive workload, time and performance pressure, reduced autonomy, lack of scope for action, lack of or insufficient positive feedback, insufficient appreciation and lack of recognition, conflict-ridden teamwork (within the team and with supervisors), information overload, constant interruptions at work, job insecurity, tasks that conflict with one’s own values, intensive emotional labour, early career stage, increased workload and poor work-life balance (Murali et al., 2018; Reichhart and Pusch, 2023). There is a clear link between burnout and various physical illnesses, including cardiovascular diseases such as arterial hypertension, coronary heart disease and heart attacks (Adebayo et al., 2023; John et al., 2024). The overall prevalence rate of burnout in Germany is estimated to be between 4% and 6% (Stöbel-Richter et al., 2013; Maske et al., 2016).

### 1.1 The autonomic nervous system in stress analysis

Several studies were analysed to investigate possible biological mechanisms that influence the risk of cardiovascular disease in people experiencing burnout and work-related stress (Adebayo et al., 2023; John et al., 2024). One of the most frequently proposed mechanisms underlying the development of burnout is autonomic nervous system (ANS) dysregulation, characterised by persistently elevated sympathetic activity and reduced parasympathetic (vagal) activity. Chronic stress leads to an imbalance in the ANS, thereby exacerbating processes of physical and psychological exhaustion. In the long term, the persistent overactivation of the sympathetic nervous system and reduction in parasympathetic activity leads to exhaustion, sensory overload and loss of function, as the ANS loses its ability to regulate itself and switch flexibly between activation and recovery. The organism is no longer able to maintain its homeostasis, i.e. its internal balance. This dysregulation of the ANS contributes to the core symptoms of burnout, characterised by emotional exhaustion due to chronic, persistent sympathetic activation; cognitive impairments (e.g. attention and memory impairments) due to reduced vagal modulation; depersonalisation and withdrawal as a result of neurobiological overload; and somatic complaints (e.g. palpitations, gastrointestinal complaints, tension headaches). Various theoretical models attempt to explain burnout scientifically through autonomic dysregulation mechanisms. Examples of these models include the allostatic load model (McEwen and Stellar, 1993; McEwen and Wingfield, 2003; McEwen, 2006), the neurovisceral integration model (Thayer and Lane, 2000; Thayer et al., 2009), the polyvagal theory (Porges, 2001, 2007), the Job Demand-Control-Support Model (Karasek, 1979; Karasek and Theorell, 1990), the Hypothalamic-Pituitary-Adrenal (HPA) Axis Dysregulation Model (Kirschbaum and Hellhammer, 1989; Heim et al., 2000; Sapolsky et al., 2000), as well as the Conservation of Resources model (Hobfoll, 1989, 2001). Although these models emphasise different levels - biological, physiological, psychological, social and behavioural - they share many common features, including chronic overactivation of the stress system, the loss of autonomic flexibility, the exhaustion of regenerative mechanisms, and an increasing vulnerability to burnout. Consequently, in medicine, burnout is not viewed merely as a purely psychological phenomenon, but as a multifactorial stress dysregulation syndrome.

### 1.2 Heart rate variability as an objective indicator of physical exertion

HRV is one of the few objective indicators of stress. It reflects processes such as long-term overactivation of the sympathetic nervous system, reduced vagal activity and impaired recovery capacity. Therefore, it can provide insights into the (over)burdening of the ANS. Several studies have shown that acute (Delaney and Brodie, 2000; Lin et al., 2001; Sgoifo et al., 2003; Hamer and Steptoe, 2007) and chronic psychosocial stress (Chandola et al., 2010; Lucini et al., 2005; Vrijkotte et al., 2000; Zanstra et al., 2006) is associated with reduced HRV (Thielmann et al., 2023). Low HRV has been linked to a variety of somatic (Thayer and Lane, 2007; Thayer et al., 2010; Koenig et al., 2016) and mental health conditions (Kawachi et al., 1995; Thayer and Sternberg, 2006; Kemp and Quintana, 2013; Chalmers et al., 2014; Sgoifo et al., 2015). It is likely that individuals suffering from burnout exhibit reduced HRV as a consequence of long-term stress. However, there are very few publications on HRV measurements in burnout patients, and these sometimes provide contradictory results (Lennartsson et al., 2016; Wekenborg et al., 2019; Lo et al., 2020; Thielmann et al., 2021; Wekenborg et al., 2022; Rubio-López et al., 2025). For example, Teisala et al. (Teisala et al., 2014) reported an association between burnout symptoms and HRV in healthy men. Lennartson et al. (2016) studied patients with and without clinical burnout, as well as a healthy control group. They found that, with the exception of the LF/HF ratio, all HRV parameters were lower in patients with clinical burnout than in the group without clinical burnout and the healthy control group. This difference was greater between the patients and the healthy control group than between the patients and the group without clinical burnout. Low HRV in burnout patients could represent a link to associated health impairments, since low HRV reflects low parasympathetic activity and thus low regenerative activity (Lennartsson et al., 2016). Another study also reported reduced HRV in patients with burnout (Vente et al., 2015). However, the study by Zanstra et al. found no differences in HRV between burnout patients and control subjects (Zanstra et al., 2006). Similarly, Thielmann et al. were unable to confirm an association between lower HRV and the three health-impairing manifestations of the three burnout dimensions (Thielmann et al., 2021). It should be noted, however, that this study examined healthy participants from a screening study in whom burnout had not been clinically diagnosed. Further research is required in this area.

### 1.3 Derivation of the research question in the context of interaction-based work

This study examines the relationship between HRV and the risk of burnout among healthy employees in professions involving interaction work. According to Böhle and Weihrich, 2020, interaction work is defined as work involving interaction with people, requiring both emotional and cognitive skills (Böhle and Weihrich, 2020). Interaction-based work represents a specific type of work-related stress. It is characterised by the continuous regulation of one’s own emotions as well as the immediate engagement with the emotions, expectations and judgements of others. Employees in interaction-intensive roles often have to deal with so-called ‘emotional dissonance’ when they are required to show emotions that do not reflect how they actually feel. This form of emotion regulation is associated with increased psychophysiological stress (Tisch et al., 2020; Rother et al., 2025) and may result in sustained activation of the ANS. Interaction-based work is characteristic of healthcare, service and social professions - that is to say, sectors in which interaction with customers, patients, clients or pupils plays a key role (Rother et al., 2025). Such activities are associated with an increased risk of developing burnout (Da Jeung et al., 2018). Furthermore, jobs that involve interaction are often characterised by unpredictability, social conflicts, frequent interruptions, and a high level of responsibility for others´ well-being (Wehrmann, 2023). Such demands can lead to reduced autonomic flexibility and impaired recovery processes. Against this background, HRV appears to be a particularly suitable objective marker of autonomic regulatory capacity for detecting the early consequences of stress in employees engaged in interaction-based work.

The aim of this study was to investigate the risk of burnout among employees in various occupational groups with a high proportion of interaction-based work and to analyse the associations between burnout dimensions (as a measure of subjective stress) and HRV (as an indicator of objective stress). The following hypothesis was formulated:

Participants who score highly on the dimensions of the Maslach Burnout Inventory or an increased risk of burnout exhibit lower HRV than participants who score normally or low scores on the burnout dimensions or a low or no risk of burnout.

## 2 Materials and methods

To address the research question of this cross-sectional study, existing data from various studies conducted by the research group were utilised. Data from the Maslach Burnout Inventory (MBI) questionnaire, as well as 6-hour electrocardiogram (ECG) and 24-hour ECG N-N interval data, were derived from three studies with comparable study designs conducted between July 2013 and April 2019. All these studies examined participants in occupations characterised by a high degree of interpersonal interaction. Prior to their involvement, detailed verbal and written information was provided to the participating subjects, and their consent was obtained. The three studies, which involved different occupational groups (bank employees, childcare workers and medical assistants), were conducted using an identical study design; the recruitment procedures were comparable, and in each case ‘convenience sampling’ was used. Seasonal variations can be ruled out, as the study periods were comparable. On the basis of these conditions, the studies were considered comparable. Approval for the conduct of the studies had been granted by the relevant ethics committee in each case (bank employees: Reg. No. 63/13, medical assistants: Reg. No. 67/13 and childcare workers: Reg. No. 40/17).

### 2.1 Sample

The inclusion criteria for participation in the above individual studies were that participants were of working age (18 and 67 years old) and had at least one year’s experience in the relevant profession. For the present analysis, data from 379 participants across the three studies were utilised. Participants were assigned to one of three different professions in the fields of banking (n = 102), childcare (n = 216) or medical assistants (MA) (n = 61).

In order to account for known factors influencing reduced or increased HRV in the present analysis, the exclusion criteria for participant selection presented in **Table 1** were considered during the data evaluation. These criteria were based on the AWMF S2k guideline ‘Use of heart rate and heart rate variability in occupational medicine and occupational science’ (Sammito et al., 2024).

**Table 1:** Exclusion criteria for the study.

With regard to…	Exclusion criteria
Diagnoses	Chronic alcohol consumption
	Cardiovascular diseases
	Elevated body mass index (BMI) or metabolic syndrome (abdominal obesity, hypertension, insulin resistance, hypertriglyceridaemia)
	Mental or neurological disorders
	Use of antidepressants (e.g. selective serotonin reuptake inhibitors)
	Smoking
	Metabolic disorders, including endocrine diseases such as diabetes and thyroid disorders
	Arterial hypertension treated with beta-blockers, L-type Ca^2+^channel blockers or calcium antagonists
	Condition following coronary stent implantation
	Condition following a heart attack, condition following a stroke
	Current pregnancy
Duties	Shift work (including night shifts)
ECG recording	Supraventricular extrasystoles >1% in the HRV measurement, ventricular extrasystoles >1% of the total number of heartbeats
	Recording duration too short (<22 h)

**Figure 1:**
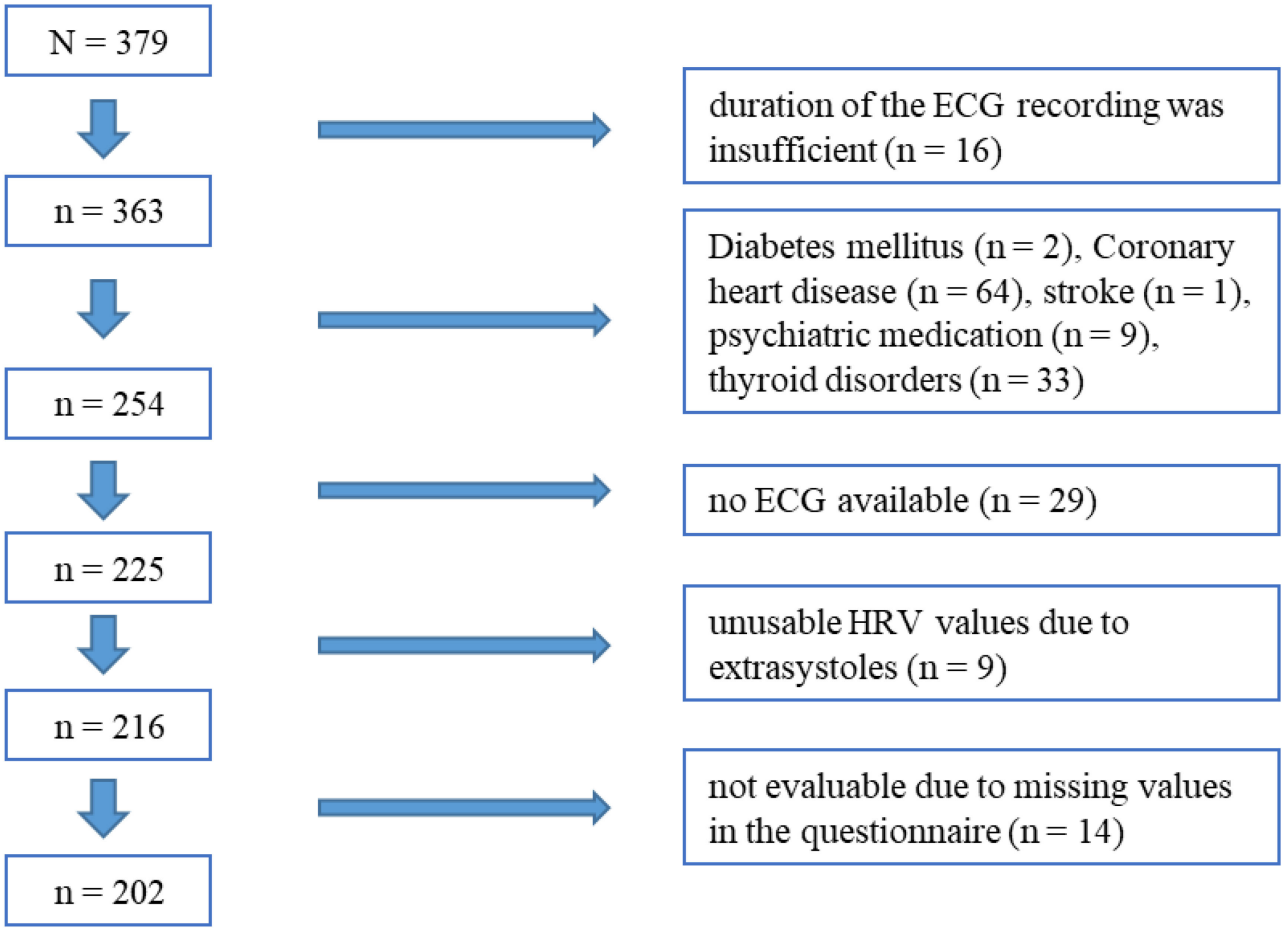
Flowchart for subject exclusion.

As a result, 134 of the 379 participants had to be excluded from the analysis, along with a further 29 participants because no ECG was available for them, and 14 because information was missing from the questionnaires. Ultimately, the present analysis could evaluate 202 participants. **Figure 1** shows the corresponding flowchart for participant inclusion.

### 2.2 Methodology

#### 2.2.1 Maslach burnout inventory questionnaire

Subjective stress was assessed by measuring the prevalence of burnout risk using the MBI (Maslach and Jackson, 1981; Schaufeli et al., 1996). This questionnaire consists of 16 items, which are assigned to the three dimensions of burnout (emotional exhaustion, cynicism/depersonalisation and personal accomplishment). The items are presented to the respondent in the form of short statements about personal feelings and attitudes. Each statement is rated on a seven-point scale, and the respondent is asked to indicate how frequently the described symptoms occur. Responses range from 0 (‘never’) to 6 (‘daily’). The mean is calculated from the scores of the variables belonging to a given dimension, and this serves as the basis for assessing the severity of that dimension. The severity scores for each burnout dimension are presented in the results table.

In line with the importance of the three burnout dimensions, Kalimo et al. (2003) also developed an algorithm to calculate the MBI total score and classify its severity. This allows the overall burnout risk to be assessed rather than each dimension being evaluated individually. Participants are categorised into one of three outcome groups: ‘no burnout’, ‘some burnout symptoms’ and ‘burnout risk’.

Kalimo’s classification of burnout risk is a practical and useful tool for the early detection of burnout risks – particularly in organisations. However, it is rarely used because it is not as well established internationally as the MBI itself, and there is no scientific validation based on larger sample sizes. It is more of a global preventive score, which should be tested for the specific setting by incorporating HRV. As burnout is a multidimensional construct, this study attempts to identify individual and global physiological correlates using HRV as an objective stress parameter.

#### 2.2.2 HRV - assessment of objective stress levels: HRV analysis

HRV reflects the physiological fluctuations in heartbeats and can be used as an objective marker for assessing sympathetic and parasympathetic activity of the ANS. HRV is based on the measurement of the successive intervals between individual heartbeats, known as RR intervals, within the QRS complex of the ECG. A long-term ECG device of the MT-101 type (2-channel recording with a sampling frequency of 1000 Hz; Schiller, Baar, Switzerland) was used to record the RR intervals in all three studies. The quality criteria for the subsequent analysis of the corresponding HRV parameters were taken into account when recording the RR intervals.

The analysis of the ECG data with regard to any (supraventricular) extrasystoles was carried out by clinical staff, who assessed the evaluability of the ECG data using the Medilog Darwin2 programme (Schiller, Baar, Switzerland). To ensure the validity of the HRV analysis, any extrasystoles present in the recordings had to be identified and removed (Sammito and Böckelmann, 2015). Recordings with an extrasystole rate exceeding 1% were excluded from the study. Subsequent HRV analysis was performed using the Kubios HRV Premium software, version 3.4.3 (University of Kuopio, Finland). An artefact correction setting (‘custom’ with ‘0.3’) and a trend component using the ‘smooth-priors’ method with ‘Lambda = 500’ were employed. To perform the spectral analysis, the settings for Fast Fourier Transformation in Kubios were adjusted to a window width corresponding to the duration of the recording, i.e. 21,600 s (for the 6-hour intervals), and a window overlap of 50%.

The subjects examined in this study are engaged in occupations whose demands are primarily characterised by psychological factors. The interpretation of this study therefore focuses on the following HRV parameters:

- from the time domain: MeanRR (distance between two R-waves), SDNN (standard deviation of all normal RR intervals), RMSSD (root mean square of successive RR interval differences), pNN50 (proportion of all pairs of successive RR intervals differing by more than 50 ms),
- from the frequency domain: LF (power spectral density in ms² in the frequency range 0.04–0.15 Hz), HF (power spectral density in ms² in the frequency range 0.15–0.4 Hz), LF n.u. (Low frequency normalised unit: corresponds to LF/(TP-VLF) * 100 in the frequency range 0.04–0.15 Hz), HF n.u. (High frequency normalised unit: corresponds to HF/(TP-VLF) * 100 in the frequency range 0.15–0.4 Hz), LF/HF (ratio of the spectrum in LF to the spectrum in HF) and
- from the non-linear domain: SD2 (standard deviation of the point distances relative to the major axis of the ellipse in ms).

Based on recent studies regarding of individual HRV parameters, this study does not consider SD1 separately, as its clinical and mathematical significance is identical to that of the RMSSD, which was also measured (Ciccone et al., 2017).

The HRV parameters included here – RMSSD, pNN50 and HF – are indicators of parasympathetic activity. However, there is disagreement regarding the interpretation of the LF component of HRV. While some researchers regard LF as a marker of sympathetic activity, most believe that it reflects fluctuations in both sympathetic and vagal activity (Berntson et al., 1997). The HRV parameters SDNN and SD2 reflect the activity of the sympathetic and parasympathetic nervous systems. However, there is no clear correlation with the LF/HF parameter. ‘Low HRV’ refers to a reduced expression of HRV parameters that reflect parasympathetic activity. The logarithmic transformation was not applied because (a) in addition to LF n.u. and HF n.u., the absolute values LF [ms²] and HF [ms²] were also plotted, and (b) the data did not need to be normally distributed, as robust statistical methods were used.

Analysis of the data sets consisted of three analysis periods: A six-hour interval during the waking period on the working day (10:00–16:00), a six-hour interval during sleep (23:00–05:00 the following day), and a 24-hour interval covering the entire measurement period from when the ECG device was attached. We selected the time intervals for the daytime phase so that all occupational groups were working during this time slot and for the nighttime phase, because during this time all participants normally are at sleep.

### 2.3 Statistics

Statistical calculations were performed on the data from the questionnaires and the HRV analysis using IBM SPSS Statistics (Version 26, IBM, Armonk, New York, USA). Descriptive statistics were used to determine the means (M), standard deviations (SD), medians and ranges.

To test the variables for normal distribution, the Kolmogorov-Smirnov goodness-of-fit test was performed, with the result that a normal distribution cannot be assumed for all HRV parameters, the means of the burnout dimensions, or age and body mass index (BMI). Subsequently, non-parametric tests were used for data analysis, —specifically the Kruskal-Wallis test for comparisons of independent samples of metrically or ordinally scaled variables—followed by the Bonferroni test for pairwise comparisons. The significance level was set at p < 0.05. A correction for multiple testing was applied to the entire set of analyses.

The following Spearman’s correlation coefficients were used to analyse the correlations, based on the work of Kuckartz et al. (2013): no correlation (0.0 < ρ < 0.1), low correlation (0.1 < ρ < 0.3), moderate correlation (0.3 < ρ < 0.5), high correlation (0.5 < ρ < 0.7) and very high correlation (0.7 < ρ < 1).

A covariance analysis was conducted using the general linear model to examine the influence of the covariates ‘gender’, ‘age’ and ‘BMI’ on HRV as the dependent variable. In our preliminary analyses of variance, we also examined occupational groups. However, no moderating effects were found. According to (Cohen, 1988), effect sizes are interpreted as follows: small effect (η² < 0.06), moderate effect (0.06 ≤ η² ≤ 0.14) and large effect (η² > 0.14).

## 3 Results

A total of 202 participants, with an average age of 41.1 ± 11.4 years (median 40.4 years, range 22–63 years), were included in the study analysis. Of these, 123 were childcare workers, 56 bank employees and 23 medical assistants (see **Table 2**). Of these, 18 were men (9%) and 182 were women (91%); two participants did not provide information on their gender.

**Table 2:** Sample composition by occupational group in the total sample, BMI, body mass index.

	Total (n = 202; 100%)	Childcare workers (n = 123; 60.9%)	Bank clerks (n = 56; 27.7%)	Medical assistance (n = 23; 11.4%)	Significance
Mean ± SD					
Parameter	Median (range)				p Kruskal-Wallis
Age	41.1 ± 11.4	40.3 ± 12.5	42.5 ± 9.6	42.6 ± 8.8	0.251
	40.4 (22.0–63.0)	37.0 (22.0–63.0)	43.0 (23.0–60.0)	40.7 (28.0–60.0)	
Years of professional experience	21.2 ± 13.2	19.9 ± 14.5	23.1 ± 10.6	23.0 ± 11.5	0.214
	21.0 (1.0–48.0)	16.0 (1.0–48.0)	23.0 (5.0–44.0)	21.0 (2.0–44.0)	
BMI (kg/m²)	25.3 ± 4.7	25.6 ± 5.0	24.8 ± 3.6	24.5 ± 5.2	0.497
	24.2 (17.0–47.7)	24.7 (17.8–47.7)	23.9 (18.8–35.8)	23.2 (17.0–36.8)	

The average BMI of the study group was 25.3 ± 4.7 (median 24.2, range 17.0–47.7). According to the WHO classification (RKI, 2017), 53.3% of participants were classified as overweight or above. Participants had been in their profession for an average of 21.2 ± 13.2 years (median 21.0, range 1.0–48.0) across all occupational groups. These occupational groups were statistically comparable (p_Kruskal-Wallis_ > 0.05) in terms of age, BMI and number of years in the profession (see **Table 2**), so all participants were included in a total sample (Total) that in the subsequent comparative analyses by burnout risk groups.

The scores for the burnout dimensions and the burnout risk according to Kalimo et al. (2003) in the total sample are presented in **Table 3**. On average, both burnout dimensions – emotional exhaustion and cynicism – were at the upper limit of low severity, while the dimension of efficacy was at the lower limit of high severity. Therefore, when considering the individual dimensions, the sample initially appeared unremarkable on average.

**Table 3:** Dimensions of the MBI-GS questionnaire and burnout risk according to Kalimo et al.(2003) in the total sample (SD = standard deviation).

Maslach burnout inventory	Total (n = 202)
Dimensions	Mean ± SD median (range)
Emotional exhaustion *(low ≤2.00; moderate 2.01–3.19; high ≥3.20)*	2.0 ± 1.34
	*1.8 (0.0–5.8)*
Cynicism/depersonalisation *(low ≤1.00; average 1.01–2.19; high ≥2.20)*	1.0 ± 1.06
	*0.6* *(0.0–4.6)*
Personal accomplishment *(low ≤4.00; average 4.01–4.99; high ≥5.00)*	5.0 ± 0.88
	*5.2* *(2.2–6.0)*
MBI-GS total score *(no burnout (symptoms never to a few times a year) 0 – 1.49; some burnout symptoms (symptoms a few times a month to almost weekly) 1.50 – 3.49; risk of burnout (symptoms several times a week to daily) 3.50 – 6.0)*	1.7 ± 1.01
	*1.7* *(0.0–4.5)*

**Figure 2:**
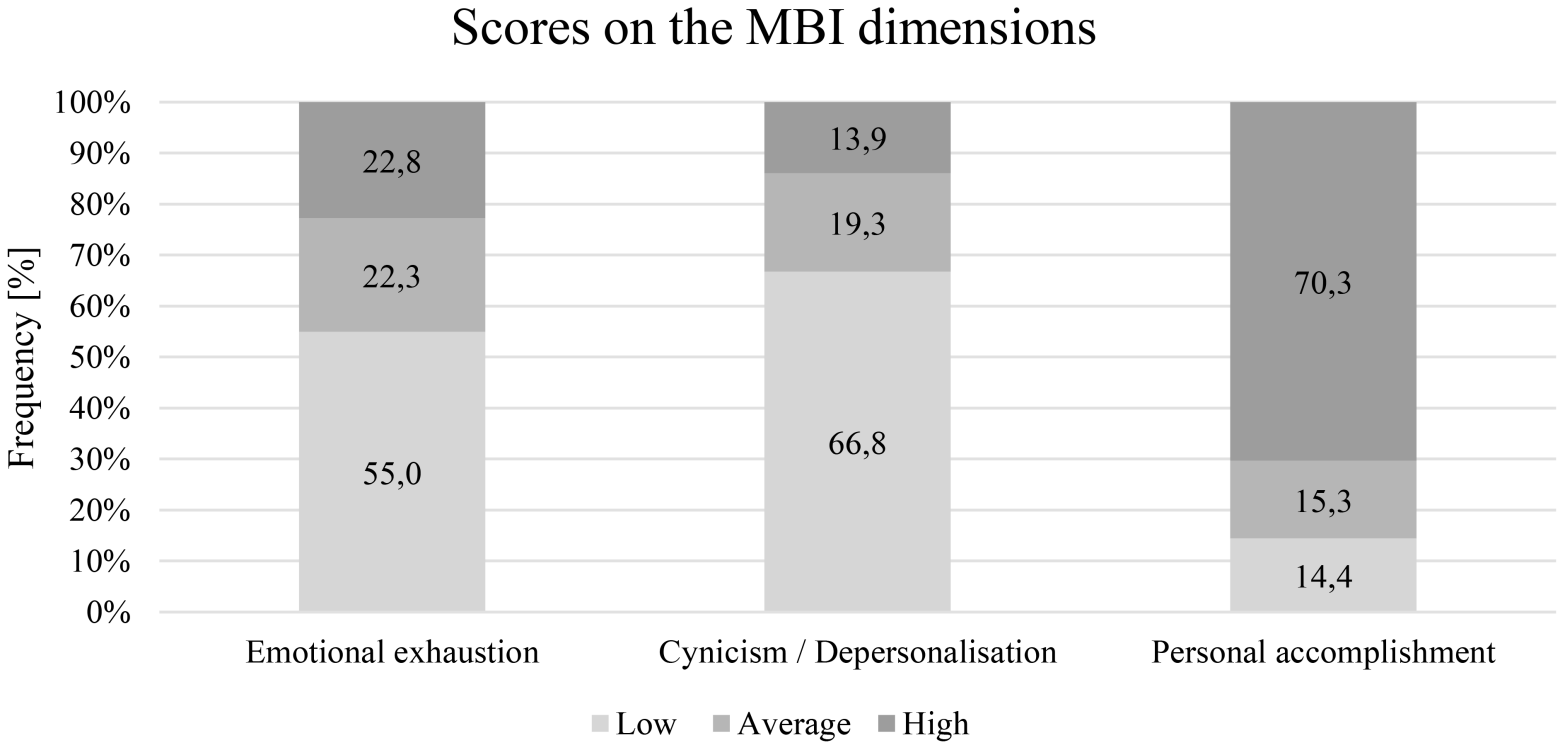
Distribution of subgroups according to levels (‘low’, ‘average’ and ‘high’; see **Table 3**) of the burnout dimensions in the total sample.

Further examination of these burnout dimensions according to the degree of severity (see **Figure 2**) revealed that 22.8% of participants exhibited very high levels of emotional exhaustion. 13.9% of participants exhibited a high level of severity in the cynicism/depersonalisation dimension. Furthermore, 14.4% of the participants in the total sample showed reduced personal accomplishment.

The average MBI total score was 1.7 ± 1.01 (**Table 3**), which is classified as “some burnout symptoms” according to the classification by Kalimo et al. (2003). For the further comparative analysis, the participants were divided into three groups according to the severity of their burnout risk, in line with the Kalimo classification: ‘no burnout’ (42.6%), ‘some burnout symptoms’ (52.0%) and “burnout risk” (5.4%).

The distribution of gender groups across the three burnout groups differed significantly (p _Pearson’s chi-square_ = 0.019). Eighty-two women (45.1%) were classified into the ‘no burnout’ group, 89 women (48.9%) into the ‘some burnout symptoms’ group and 11 women (6%) into the ‘burnout’ group. All participants in the ‘burnout’ group were female. Three male participants (16.7%) belonged to the ‘no burnout’ group and 15 (83.3%) to the ‘some burnout symptoms’ group.

Age, years of professional experience and BMI were compared across these burnout groups (see **Table 4**), with significant differences found for age and years of professional experience (p_Kruskal-Wallis_ = 0.003 and 0.006 respectively). All three groups were statistically comparable in terms of BMI (p_Kruskal-Wallis_ = 0.606). Subsequent general linear model analyses took into account age and gender, which is known to be a factor influencing HRV.

**Table 4:** Age, BMI and years in employment for the groups, based on the classifications by Kalimo et al. (2003): ‘no burnout’, ‘some burnout symptoms’ and ‘increased risk of burnout’.

		Total (n = 202)	No burnout (NB) (n = 86; 42.6%)	some burnout symptoms (SBS) (n = 105; 52.0%)		Increased risk of burnout (IRB) (n = 11; 5.4%)		Significance		
Mean ± SD							pKruskal-Wallis		pBonferroni	
Median (Range)										
Age	41.1 ± 11.4		38.1 ± 11.5		43.5 ± 10.6	42.9 ± 12.1	**0.003**			**NB-SBS (0.002)**
	*40.4* *(22.0–63.0)*		*34.0* *(23.0–62.0)*		*46.0* *(22.0–63.0)*	*45.0* *(26.0–61.0)*				
Years of professional experience	21.2 ± 13.2		17.7 ± 13.7		23.8 ± 12.3	23.0 ± 14.0	**0.006**			**NB-SBS (0.004)**
	*21.0* *(0.0–48.0)*		*15.0* *(1.0–45.0)*		*25.0* *(0.0–48.0)*	*24.0* *(1.0–41.0)*
BMI	25.3 ± 4.7		25.7 ± 4.9		24.9 ± 4.4	25.7 ± 5.9	0.606			-–
	*24.2* *(17.0–47.7)*		*35.2* *(17.0–37.8)*		*24.0* *(18.8–47.7)*	*23.9* *(20.4–40.4)*

SD = Standard deviation; statistically significant p-values are in bold.

First, the HRV parameters of the participants for the respective time intervals analysed (24 hours, 6-hour day and 6-hour night) were examined across the three burnout dimensions severity groups (see Supplement). Here, only isolated significant differences in HRV parameters were found between the respective severity groups (‘low’, ‘moderate’ and ‘high’). The cynicism dimension showed significant differences across the severity levels in one of the examined parameters, MeanRR, in the 6-hour daytime interval (p_Kruskal-Wallis_ = 0.037). Analysis of the personal accomplishment dimension shows that there is a significant difference between the different severity levels for the examined HRV parameter LF (P_Kruskal-Wallis_ = 0.034).

Subsequent comparisons of HRV parameters across the three burnout groups (‘no burnout’, ‘some burnout symptoms’ and ‘burnout risk’) revealed significant differences in mean values for four HRV parameters within the 24-hour interval (HF with p_Kruskal-Wallis_ = 0.039, LF n.u. with p_Kruskal-Wallis_ = 0.009, HF n.a. with p_Kruskal-Wallis_ = 0.009 and LF/HF with p_Kruskal-Wallis_ = 0.009). In the 6-hour daytime interval, only one parameter showed a significant difference (MeanRR with p_Kruskal-Wallis_ = 0.002), while no significant differences were observed in the 6-hour night-time interval (**Tables 5**–**7**).

**Table 5:** Comparisons of HRV parameters for the 24-hour interval between the levels of burnout risk using the Kruskal-Wallis test.

		Total	Burnout risk					Significance	
		n=202	No burnout (NB) (n=86)		Some burnout symptoms (SBS) (n=105)		Increased risk of burnout (IRB) (n=11)		
Mean ± SD								pKruskal-Wallis	pBonferroni
Median (Range)									
MeanRR [ms]	775.7 ± 81.47			761.2 ± 71.16		786.14 ± 90.40	788.8 ± 47.10	0.077	-–
	774.4 (591.4–1101.5)			759.6 (626.5–949.8)		782.2 (591.4–1101.5)	793.4 (675.5–856.1)		
SDNN [ms]	143.6 ± 34.07			147.3 ± 35.94		140.5 ± 33.16	143.6 ± 26.28	0.616	-–
	143.2 (59.3–255.5)			144.5 (82.3–255.5)		140.9 (59.3–240.3)	142.4 (105.0–186.8)	-–
RMSSD [ms]	32.9 ± 15.98			35.1 ± 19.01		30.9 ± 13.22	34.2 ± 12.64	0.340
	28.3 (10.3–123.3)			28.7 (11.5–123.3)		27.5 (10.3–79.5)	33.2 (21.0–57.4)	
pNN50	9.9 ± 8.41			10.8 ± 8.96		9.0 ± 7.90	11.0 ± 8.69	0.328	-–
	7.1 (0.1–70.8)			7.6 (0.2–43.8)		6.4 (0.1–39.2)	9.9 (2.1–28.8)
LF [ms²]	1000.7 ± 695.80			1055.9 ± 728.39		961.4 ± 687.84	944.0 ± 505.18	0.621	-–
	779.8 (139.8–3884.2)			797.9 (167.9–3168.0)		757.9 (139.8–3884.2)	773.6 (335.8–2054.0)
HF [ms²]	560.5 ± 935.00			769.4 ± 1327.30		401.0 ± 419.57	449.1 ± 329.83	**0.039**	**NB-IRB (0.038)**
	324.6 (27.1–10,422.2)			391.2 (43.1–10,422.2)		287.1 (27.1–2671.8)	269.8 (192.3–1179.9)
LF n.u.	70.1 ± 11.85			66.8 ± 13.26		72.9 ± 10.30	68.7 ± 7.00	**0.009**	**SBS-IRB (0.010)**
	71.6 (22.2–91.7)			70.5 (22.1–86.4)		72.2 (41.5–91.7)	70.5 (59.7–81.8)
HR n.u.	29.9 ± 11.85			33.1 ± 13.26		27.1 ± 10.30	31.2 ± 7.00	**0.009**	**NB-SBS (0.010)**
	28.4 (8.3–77.9)			29.5 (13.6–77.8)		27.7 (8.3–58.5)	29.5 (18.2–40.2)
LF/HF	2.9 ± 1.74			2.5 ± 1.42		3.3 ± 1.94	2.4 ± 0.88	**0.009**	**SBS-IRB (0.010)**
	2.5 (0.3–11.0)			2.4 (0.3–6.4)		2.6 (0.7–11.0)	2.4 (1.5–4.5)
SD2 [ms]	201.5 ± 47.54			206.6 ± 50.03		197.4 ± 46.39	201.5 ± 36.88	0.647	-–
	200.6 (83.4–352.3)			203.0 (116.1–352.3)		198.5 (83.4–336.2)	200.0 (147.1–261.8)

SD = Standard deviation; statistically significant p-values are in bold.

**Table 6:** Comparisons of HRV parameters for the 6-hour daily interval between the categories of burnout risk using the Kruskal-Wallis test.

	Total	Burnout risk						Significance	
	n=202	No burnout (NB) (n=86)		Some burnout symptoms (SBS) (n=105)		Increased risk of burnout (IRB) (n=11)			
	Mean ± SD							PKruskal-Wallis	PBonferroni
Median (Range)									
Mean RR [ms]	708.6 ± 88.21		684.1 ± 72.46		725.6 ± 97.57		736.9 ± 60.77	0.002	**NB-SBS (0.005)**
	701.7 (518.2–1075.5)		673.8 (549.9–893.4)		718.7 (518.2–1075.5)		741.9 (629.7–864.2)		
SDNN [ms]	88.8 ± 22.58		86.8 ± 21.02		89.4 ± 24.28		97.9 ± 15.18	0.148	-–
	84.5 (44.7–177.2)		82.1 (51.5–155.6)		84.8 (44.7–177.2)		98.9 (72.0–122.7)
RMSSD [ms]	25.8 ± 10.73		25.0 ± 10.32		26.1 ± 11.20		29.1 ± 9.24	0.213	-–
	23.6 (8.9–74.1)		22.6 (8.9–63.1)		24.0 (10.1–74.1)		28.8 (16.9–50.1)
pNN50	6.4 ± 6.63		6.0 ± 6.23		6.5 ± 6.97		8.5 ± 6.58	0.33	-–
	4.2 (0.0–41.3)		4.0 (0.2–29.8)		4.2 (0.0–41.3)		7.8 (0.9–24.0)
LF [ms²]	952.0 ± 633.66		945.1 ± 614.74		958.6 ± 670.86		943.1 ± 420.62	0.838	-–
	810.3 (122.8–4233.4)		846.1 (146.9–3782.7)		782.2 (122.8–4233.4)		917.4 (344.3–1676.2)
HF [ms²]	295.2 ± 339.78		302.3 ± 380.45		283.9 ± 313.67		347.7 ± 249.60	0.277	-–
	188.1 (24.1–2407.9)		179.4 (24.1–2407.8)		198.1 (32.2–1628.5)		312.5 (78.9–933.0)
LF n.u.	78.9 ± 9.00		79.3 ± 8.61		79.0 ± 9.29		74.7 ± 8.37	0.170	-–
	79.8 (53.1–92.9)		81.4 (54.5–92.6)		79.7 (53.1–92.9)		77.4 (54.5–84.7)
HR n.u.	21.1 ± 8.96		20.7 ± 8.60		20.9 ± 9.27		25.3 ± 8.37	0.173	-–
	20.2 (7.1–46.8)		18.6 (7.4–45.4)		20.3 (7.1–46.8)		22.6 (15.2–45.3)
LF/HF	4.7 ± 2.56		4.7 ± 2.30		4.9 ± 2.81		3.3 ± 1.22	0.171	-–
	4.0 (1.1–13.0)		4.4 (1.2–12.5)		3.9 (1.1–13.0)		3.4 (1.2–5.6)
SD2 [ms]	124.1 ± 31.47		121.4 ± 29.29		125.0 ± 33.85		136.8 ± 21.19	0.142	-–
	118.1 (62.9–245.0)		115.1 (71.5–217.7)		118.8 (62.9–245.0)		138.4 (100.7–172.0)

SD = Standard deviation; statistically significant p-values are in bold.

**Table 7:** Comparisons of HRV parameters for the 6-hour night-time interval between levels of burnout risk using the Kruskal-Wallis test.

	Total	Burnout risk					Significance				
	n=202	No burnout (NB) (n=86)		Some burnout symptoms (SBS) (n=105)		Increased risk of burnout (IRB) (n=11)					
	Mean ± SD									pKruskal-Wallis	pBonferroni
Median (range)											
Mean RR [ms]	939.6 ± 109.51		935.3 ± 106.54		942.8 ± 115.30			942.0 ± 77.59	0.778		-–
	936.0 (657.2–1370.3)		906.0 (722.4–1256.8)		945.6 (657.2–1370.3)			962.2 (820.3–1058.1)			
SDNN [ms]	93.9 ± 27.71		97.3 ± 28.07		91.8 ± 28.19			88.0 ± 17.00	0.265		-–
	88.9 (39.7–195.0)		90.4 (48.4–195.0)		86.3 (39.7–194.3)			90.7 (67.2–119.6)
RMSSD [ms]	43.7 ± 26.75		49.3 ± 32.67		39.1 ± 20.35			43.7 ± 22.50	0.082		-–
	36.1 (10.1–197.2)		41.3 (11.6–197.2)		32.8 (10.1–110.7)			32.3 (19.1–79.2)
pNN50	18.4 ± 16.68		21.5 ± 18.03		15.8 ± 15.01			18.9 ± 18.13	0.079		-–
	12.8 (0.0–77.4)		18.4 (0.1–77.4)		10.4 (0.0–67.3)			8.9 (1.7–47.8)
LF [ms²]	1203.1 ± 1068.58		1318.7 ± 1220.57		1132.9 ± 956.30			969.8 ± 742.03	0.316		-–
	866.4 (131.4–8479.2)		910.9 (188.0–9479.2)		763.2 (131.4–5946.9)			738.3 (273.5–2744.6)
HF [ms²]	861.9 ± 1482.04		1159.3 ± 2097.93		647.5 ± 700.57			584.5 ± 552.40	0.087		-–
	417.7 (27.3–15,868.4)		523.6 (37.3–15,848.4)		329.0 (27.3–3863.49)			343.1 (79.8–1647.7)
LF n.u.	65.1 ± 14.15		62.2 ± 15.1		67.3 ± 13.1			65.8 ± 13.0	0.101		-–
	66.3 (26.9–89.3)		63.3 (26.9–89.3)		67.9 (27.6–89.2)			65.8 (46.8–85.7)
HR n.u.	34.9 ± 14.14		37.7 ± 15.12		32.7 ± 13.12			34.1 ± 13.00	0.102		-–
	33.7 (10.7–73.1)		36.6 (10.7–73.1)		32.0 (10.8–72.4)			34.2 (14.3–52.9)
LF/HF	2.4 ± 1.62		2.2 ± 1.48		2.7 ± 1.69			2.5 ± 1.63	0.102		-–
	2.0 (0.4–8.3)		1.7 (0.4–8.3)		2.1 (0.4–8.3)			1.9 (0.9–6.0)
SD2 [ms]	128.6 ± 36.48		132.2 ± 35.51		126.5 ± 38.37			120.0 ± 21.69	0.328		-–
	122.6 (55.6–270.4)		125.0 (66.9–242.4)		119.3 (55.6–270.4)			123.6 (93.7–160.4)

Subsequent pairwise comparisons using Bonferroni correction show that the HRV parameter HF differs significantly between the categories ‘no burnout’ and ‘increased risk of burnout’ (p_Bonferroni_ = 0.038). Furthermore, significant differences were observed in the HRV parameter LF n.u. between the categories ‘some burnout symptoms’ and ‘burnout risk’ (p_Bonferroni_ = 0.010), as well as in the HRV parameter HF n.u. between ‘no burnout’ and ‘some burnout symptoms’ (p_Bonferroni_ = 0.010) and in the HRV parameter LF/HF between ‘some burnout symptoms’ and ‘increased burnout risk’ (p_Bonferroni_ = 0.010).

In the 6-hour daily interval, a Bonferroni-corrected difference in the MeanRR parameter was demonstrated between the categories ‘no burnout’ and ‘some burnout symptoms’ (p_Bonferroni_ = 0.005) (**Table 6**).

Examining the 6-hour night-time interval shows no significant differences in burnout risk levels across the studied HRV parameters (**Table 7**).

### 3.1 Correlation and variance analysis

**Table 8** shows the Spearman’s rho correlations between HRV parameters and the burnout dimensions, as well as the MBI total score, for three measurement intervals (24 h, 6 h day, 6 h night). Overall, the correlations are predominantly weak.

**Table 8:** Spearman’s rho correlation of HRV parameters at different time intervals with three burnout dimensions and the MBI total score.

	24h				6h day				6h night			
ρ	EE	CY	PA	MBI total score	EE	CY	PA	MBI total score	EE	CY	PA	MBI total score
MeanRR [ms]	0.038	0.046	-0.087	0.043	0.114	0.109	-0.082	0.200**	-0.074	0.006	-0.086	-0.089
SDNN [ms]	-0.091	-0.070	0.083	-0.104	-0.131	-0.104	0.085	-0.206**	0.050	-0.021	0.083	0.067
RMSSD [ms]	0.032	0.007	0.096	0.020	-0.030	-0.034	0.130	-0.072	0.133	0.040	0.065	0.151*
pNN50	0.158*	0.122	-0.060	0.179*	0.180*	0.155*	-0.051	0.271**	0.058	0.097	-0.094	0.051
LF [ms²]	-0.158*	-0.122	0.060	-0.179*	-0.180*	-0.155*	0.051	-0.271**	-0.058	-0.097	0.094	-0.051
HR [bpm²]	-0.145*	-0.045	-0.012	-0.218**	-0.093	-0.049	-0.038	-0.199**	-0.119	-0.139*	0.030	-0.153*
LF n.a.	0.067	0.003	0.055	0.093	-0.102	-0.134	0.067	-0.118	0.082	-0.012	0.058	0.103
HF n.a.	-0.124	-0.145*	0.086	-0.194**	-0.175*	-0.191**	0.120	-0.245**	0.002	-0.072	0.038	-0.063
LF/HF ratio	-0.105	-0.074	-0.071	-0.128	-0.013	0.015	-0.105	0.013	-0.161*	-0.096	-0.053	-0.188**
SD2 [ms]	-0.034	0.059	-0.088	-0.076	0.054	0.100	-0.071	0.027	-0.039	-0.095	0.062	-0.059

red, significant negative correlation with 0.1<ρ<0.3, green, significant positive correlation with 0.1<ρ<0.3; EE, emotional exhaustion; CY, cynicism; PA, personal accomplishment; *p < 0.05, **p < 0.01.

The burnout dimensions emotional exhaustion and cynicism/depersonalisation show the most correlations in the 6-hour daytime interval, followed by the 24-hour interval (**Table 8**). In the 6-hour night-time interval, only a few significant correlations were found. The LF dimension of the MBI shows no significant correlation with any of the HRV parameters examined in any time interval. According to Kalimo et al. (2003) the different time intervals of the MBI total score exhibit a similar distribution pattern of significant correlations: a slightly higher number of correlations is found in the 6-hour daytime interval, followed by the 24-hour and 6-hour night-time intervals.

Significant negative correlations were found over the 24-hour interval between the HRV parameter LF and the burnout dimension of emotional exhaustion (ρ = −0.158; p < 0.05) as well as with the MBI total score (ρ = −0.179; p < 0.05). The HRV parameter HF also correlated negatively with emotional exhaustion (ρ = −0.145; p < 0.05) and with the MBI total score (ρ = −0.218; p < 0.01). In contrast, pNN50 showed a significant positive correlation with emotional exhaustion (ρ = 0.158; p < 0.05) and with the MBI total score (ρ = 0.179; p < 0.05). No significant correlations were found for the remaining parameters.

The strongest associations occurred within the 6-hour daytime interval. The MBI total score correlated positively with both the mean RR interval (MeanRR: ρ = 0.200; p < 0.01) and pNN50 (ρ = 0.271; p < 0.01). At the same time, significant negative correlations were observed with SDNN (ρ = −0.206; p < 0.01), LF power (ρ = −0.271; p < 0.01) and HF power (ρ = −0.199; p < 0.01). Furthermore, pNN50 correlated positively with emotional exhaustion (ρ = 0.180; p < 0.05) and with cynicism/depersonalisation (ρ = 0.155; p < 0.05). Negative correlations were also observed between LF power and emotional exhaustion (ρ = −0.180; p < 0.05), and between LF power and cynicism/depersonalisation (ρ = −0.155; p < 0.05).

During the 6-hour night phase, the correlations were generally less pronounced. Significant negative correlations were found between HF power and cynicism (ρ = −0.139; p < 0.05) as well as between the LF/HF ratio and emotional exhaustion (ρ = −0.161; p < 0.05) and the MBI total score (ρ = −0.188; p < 0.01). A significant positive correlation was observed between RMSSD and personal accomplishment (ρ = 0.151; p < 0.05).

**Table 9** shows the results of the analysis of variance for the HRV parameters over a 24-hour period, taking into account the influencing factors of age, gender, BMI and burnout risk group. According to the general linear model, age has a moderate effect on the parameters MeanRR, LF and HF, and a large effect on the HRV parameters RMSSD and pNN50 from the time domain, as well as LF n.s. and HF n.s. from the frequency domain. Gender has a moderate effect on LF and a small effect on MeanRR. Body mass index (BMI) showed no significant influence on the examined HRV parameters. The effect sizes were consistently very small (η² ranging from 0.000 to 0.016). Membership of the individual burnout risk groups and BMI had no effect. Burnout risk alone had no effect on the HRV parameters. Effect sizes were generally small, ranging from η² = 0.000 to 0.030. The results of this model highlight the earlier finding that the significant differences in the HRV parameters are not due to the burnout risk groups, but to the well-known phenomenon that HRV is affected by age and gender.

**Table 9:** Analysis of variance of the 24-hour HRV interval for the total sample.

HRV parameters	Model			Age		Gender		BMI		Burnout risk group	
	Coefficient of determination (R²)	p	η²	p	η²	p	η²	p	η²	p	η²
**MeanRR [ms]**	0.079	**0.006**	0.112	**< 0.001**	0.078	**0.032**	0.033	0.172	0.014	0.585	0.008
**SDNN [ms]**	0.023	0.150	0.057	0.123	0.170	0.132	0.017	0.161	0.014	0.835	0.003
**RMSSD [ms]**	0.178	**< 0.001**	0.207	**< 0.001**	0.173	0.305	0.008	0.343	0.004	0.688	0.005
**pNN50**	0.182	**< 0.001**	0.211	**< 0.001**	0.178	0.248	0.008	0.385	0.006	0.538	0.009
**LF [ms²]**	0.182	**< 0.001**	0.211	**< 0.001**	0.105	**< 0.001**	0.010	0.531	0.003	0.980	0.000
**HF [ms²]**	0.086	0.004	0.119	**< 0.001**	0.090	0.807	0.097	0.970	0.000	0.541	0.009
**LF n.u.**	0.203	**< 0.001**	0.231	**< 0.001**	0.175	0.088	0.000	0.136	0.016	0.266	0.019
**HF n.u.**	0.203	**< 0.001**	0.231	**< 0.001**	0.175	0.089	0.021	0.137	0.016	0.266	0.019
**LF/HF**	0.177	**< 0.001**	0.206	**< 0.001**	0.140	0.067	0.024	0.194	0.012	0.125	0.030
**SD2 [ms]**	0.020	0.171	0.055	0.158	0.015	0.130	0.017	0.145	0.016	0.843	0.003

Effect sizes: small effect (η² < 0.06, no colour coding), moderate effect (0.06 ≤ η² ≤ 0.14, colour coding light green), large effect (η² > 0.14, colour coding dark green). Significant values are highlighted in bold; BMI, Body Mass Index.

## 4 Discussion

The present study examines the relationship between burnout (as a measure of subjective stress) and heart rate variability (as an indicator of objective stress) in individuals with varying levels of burnout risk. Overall, only weak associations were found between burnout risk and HRV parameters in healthy employees with high levels of interactive work. Meanwhile, sociodemographic factors, particularly age, were found to have significantly stronger influence on autonomic regulation.

When the differences between the burnout risk levels (no burnout, some symptoms and burnout) and the examined HRV parameters were considered, only a few significant differences between the levels were observed for some HRV parameters. These differences were only found in the 24-hour period (four significant correlations: HF, LF n.s., HF n.s. and LF/HF) and the 6-hour day-night interval (MeanRR). This effect disappears when co-factors that influence HRV, such as age and gender, are taken into account.

The nocturnal phase is characterised by dominant parasympathetic activity, enabling physiological recovery of the organism (Sammito and Böckelmann, 2016). Corresponding HRV measurements show elevated values in healthy individuals; conversely, reduced nocturnal HRV parameters indicate impaired regenerative capacity (May et al., 2018). The present study reveals significant differences in individual parameters between participants with an increased risk of burnout and those without across both the 24-hour and the 6-hour daytime intervals. Although significant associations with burnout dimensions were found for individual HRV parameters, these were predominantly weak and occurred primarily during the daytime. This pattern suggests that subjective stress experienced during interaction-intensive activities may primarily be reflected in situational, work-related regulatory processes, while nocturnal recovery mechanisms appear to be less clearly impaired.

At the same time, the lack of a significant influence of burnout risk groups within the 24-hour period suggests that autonomic dysregulation in non-clinically apparent burnout is rather strongly affected by individual predispositions. Given this, while HRV remains a promising objective marker of work-related stress, its ability to differentiate between early stages of burnout in healthy working populations should be viewed with caution and with due consideration of moderating factors such as age, stress levels and the time of measurement.

### 4.1 Discussion of the study results in the light of the international literature

The associations between HRV and burnout have been addressed in several studies in the literature, although the results do not show a clear trend (Lennartsson et al., 2016; Lo et al., 2020; Thielmann et al., 2021; Wekenborg et al., 2022). The lifetime prevalence of burnout in the general population is 4.2%, making it a significant issue in today’s working world (Maske et al., 2016). This study identified a high risk of burnout in 5.3% of participants. However, this is not directly comparable with the figures from the study by Maske and co-authors.

Most studies have examined the HRV of patients with overt burnout (Lennartsson et al., 2016; Wekenborg et al., 2019). This study found that a high score on emotional exhaustion is a predictive factor for a reduction in HRV (after twelve months), but not for the MBI total score (Wekenborg et al., 2019). Both studies demonstrate a significant yet complex relationship between the autonomic regulation of the cardiovascular system and burnout symptoms. However, measurement duration and conditions must considered as methodological limitations. Kanthak et al (Kanthak et al., 2017). found similar results regarding the role of the burnout dimension ‘emotional exhaustion’ when investigating autonomic dysregulation in burnout and depression. They found that emotional exhaustion correlates negatively with the HRV parameter RMSSD (Kanthak et al., 2017). Other studies, such as that by Traunmüller et al., found no relationship between HRV and burnout. In this study, associations with reduced HRV were found among the individuals with burnout symptoms who were examined. However, this association could not be confirmed beyond doubt by further analyses. The MBI-GS was used to assess burnout (Traunmüller et al., 2019).

It should be noted that none of the participants in this study had been medically diagnosed with burnout. This could explain why no significant correlations of HRV parameters were found during the night-time interval. HRV analysis is claimed to be a suitable preventive screening tool. It is possible that this could be observed particularly during the night, as HRV analysis is not influenced at night by everyday activities, which are typically associated with sympathetic activity. In participants without a diagnosed case of burnout, however, associations with reduced HRV parameters were found, which could indicate the potential benefits of a preventive intervention. The observed correlations largely disappear once demographic covariates are taken into account.

Clearly, the results of the various studies were obtained under conditions that, in some cases, were very different. For instance, the studies by Wekenborg et al. and Lennartsson et al. are characterised by comparatively short measurement periods (335s and 300s respectively) (Lennartsson et al., 2016; Wekenborg et al., 2019). As standardised periods, these measurement scenarios thus allowed for better comparability of the results. They are well suited to study designs aimed at a direct response of the organism to a strictly time-limited event. However, such short, standardised measurement periods make it difficult to realistically reflect an individual´s HRV in various everyday situations. For this reason, in this study we opted for a long-term measurement over 24 hours and, based on this, a 6-hour window. Whilst this does have its advantages – for example, it allows night-time measurements to be taken without waking the participants – long-term measurements also have the disadvantage that everyday activities can influence the analyses. Furthermore, the frequency-based HRV parameters in particular were originally validated primarily for short-term measurements lasting 5 minutes (*Heart rate variability: standards of measurement, physiological interpretation and clinical use.* Task Force of the European Society of Cardiology and the North American Society of Pacing and Electrophysiology, 1996). As this applies to all groups of participants across all burnout categories, the effect can be classified as reduced for the purposes of this study, but it does make comparisons with short-term measurements more difficult. Therefore, it is necessary to assess in advance of the study whether a high degree of inter-individual comparability of the results is desired, in which case a standardised (shorter) study setting should be planned, or whether a long-term recording focusing on individual daily routines is likely to be more promising.

It is worth noting here the widespread use of the MBI-GS to assess the risk of burnout. The studies considered here used the Maslach Burnout Inventory (MBI-GS), the Copenhagen Burnout Inventory (CBI) and the Shirom-Melamed Burnout Questionnaires (SMBQ) were used. While the inconsistent use of the various burnout assessment instruments makes it difficult to compare the results; the diversity of these instruments ultimately improves quality provided that the questionnaires have consistently high internal validity. The results of the SMBQ are comparable with those of the MBI-GS due to their established internal validity (Lundgren-Nilsson et al., 2012). The fundamental comparability of the CBI with the results of the MBI-GS is also considered to be well-established (Winwood and Winefield, 2004). At present, it can be said that the MBI-GS is regarded as the ‘gold standard’ for assessing burnout (Williamson et al., 2018), although this is not without criticism regarding the quality of its internal validity (Burisch, 2014).

In summary, it is notable that a wide range of research methodologies have been employed in studies on this topic. Both long-term analyses of HRV (e.g. 24-hour intervals) and short-term analyses are used. When considering such studies, potential sources of error should generally be taken into account. For example, subjective biases can arise from self-assessment of stress levels (e.g. by completing questionnaires). Furthermore, even objective methods of measuring stress can be subject to various sources of error. Controlling for factors that influence HRV (e.g. conducting medical examinations rather than basing information purely on medical history) increases the validity of the physiological stress parameters. Therefore, a sensible compromise lies in collecting improved-quality data combined with a study design that conserves resources.

### 4.2 Limitations

The sample is highly imbalanced in terms of occupational groups and only represents a small proportion of the labour market. Compared to studies limited to a single occupational group (e.g (Violanti et al., 2018)), the overall sample exhibits greater heterogeneity.

Therefore, the results cannot therefore be generalised, as the sample was predominantly drawn from the Magdeburg area using ‘convenience sampling’. Furthermore, childcare workers, bank employees and medical assistants were only included if their institutions were willing to participate in the respective research studies. This selection strategy may generate systematic biases, for example because institutions with precarious working conditions or low staff-to-child ratios may decline to participate in the study for capacity reasons or, conversely, they may be particularly interested in participating in prevention measures for motivational reasons. Consequently, the external validity of the findings is limited.

In the sample, only eleven individuals were classified as being at increased risk of burnout and all were female, which severely limits the generalisability of the findings on burnout. Given the large number of HRV parameters analysed, this appears insufficient to enable reliable multiple comparisons between the three groups. A larger and more balanced sample would therefore be recommended for future studies in order to achieve greater stability in the estimates, sufficient statistical power and good reliability regarding any differences between the groups. Furthermore, the ‘healthy worker effect’ was present. As individuals who had already left the workforce due to illness could not be surveyed, meaning that only healthy, able-bodied employees were included. This constitutes a potential selection bias (Baillargeon, 2001).

The sample under investigation comprises 90.6% women and only 9.4% men. This gender imbalance can be attributed to the significant representation of nursery teachers (n = 123, corresponding to 60.9% of the sample), a profession with a high proportion of female employees. This group, which is the largest in the studied sample, has a particularly high proportion of women (96.8% within the sub-sample), comparable to the representation of this occupational group in the Federal Republic of Germany (92.6%) (Statistisches Bundesamt, 2021). The sample is gender-imbalanced, which meant that a gender-specific influence on the HRV parameters was to be expected. Furthermore, the gender distribution across the different burnout groups was not even; the relatively small group of people with an ‘increased risk of burnout’ consisted entirely of female participants. Women show lower MeanRR and SDNN values, lower total power, higher HR, lower LF and LF/HR. For this reason, gender, which is known to be a factor influencing HRV, was included as a covariate in the analysis of variance of the HRV parameters (Koenig and Thayer, 2016; Sammito and Böckelmann, 2016; Sammito et al., 2024). Taking this into account, the model showed that burnout risk had no effect on any of the HRV parameters.

Another limitation of the present study is the fact that potential medical conditions were identified through questioning the participants, which may have resulted in their exclusion from the present study. However, unknown medical conditions (e.g. thyroid disorders) could influence HRV and thus create selection bias. Participants with a high number of extrasystoles or other arrhythmias were subsequently excluded.

A key starting point for optimising the design of the present study lies in the systematically recording and considering of any internal, subject-related confounding factors. In the current experimental design, factors that could potentially modulate HRV, such as smoking habits, alcohol consumption and physical activity, were not examined in sufficient depth in the statistical analyses, since the three occupational groups were statistically comparable with regard to these variables. However, as these variables have been shown to modulate heart rate variability (Fagard et al., 1999), it is essential that they are consistently collected and rigorously statistically controlled in order to enhance the internal validity and interpretability of HRV measurements in future studies. Our study has a cross-sectional design; therefore, it does not allow us to draw causal conclusions about the relationship between burnout symptoms and autonomic regulation. Furthermore, the greater influence of age on the results must be taken into account here.

### 4.3 Outlook for practice

The issue of burnout in the workplace and its effects on HRV is a relevant topic that should continue to be addressed in the future and investigated further using appropriate research designs (Aronsson et al., 2017). From a preventive medicine perspective, it is important to identify employees at risk of burnout at an early stage in order to be able to initiate preventive measures. Undoubtedly, every occupational group faces its unique individual challenges. Therefore, future research should investigate how specific groups can be relieved of stressors or how the resources of employees in these groups can be strengthened to prevent burnout. In general, a possible measure is to intervene or educate employees about health risks in cases where stress management strategies are inadequate or where there are early signs of burnout. In order to provide employees with relevant preventive measures, it is necessary to raise awareness of the issue of burnout among both employees and managers.

## Data Availability

The raw data supporting the conclusions of this article will be made available by the authors, without undue reservation.
